# Multiple Severe Toxicities of L-Asparaginase and Their Innovative Management during Induction Therapy of Acute Lymphoblastic Leukemia in an Adult Patient

**DOI:** 10.1155/2019/9086570

**Published:** 2019-11-20

**Authors:** Guillaume Beziat, Suzanne Tavitian, Muriel Picard, Stanislas Faguer, Christian Recher, Françoise Huguet

**Affiliations:** ^1^Service d'Hématologie Clinique, Institut Universitaire du Cancer Oncopole, Toulouse, France; ^2^Réanimation et Soins Intensifs, Institut Universitaire du Cancer Oncopole, Toulouse, France; ^3^Secteur de Réanimation de l'Unité de Transplantation d'Organes, CHU Rangueil, Toulouse, France

## Abstract

L-asparaginase is a key chemotherapeutic agent in acute lymphoblastic leukemia (ALL). It is also known for multiple and severe specific toxicities, without consensual management. We report the case of a 51-year-old man treated with L-asparaginase for recently diagnosed T-cell ALL. During the treatment, he developed a coma due to multifactorial diffuse cerebral edema, by hepatic encephalopathy, cerebral venous thrombosis, and hyperammonemia, all linked to toxicity of L-asparaginase. Specific and innovative treatments were employed to manage these toxicities: supplementation with L-carnitine, thiamine, and pyridoxine for hepatic toxicity, perfusion of sodium benzoate to decrease ammonemia, and extrahepatic albumin-based dialysis sessions, along with anticoagulation. The patient improved within two weeks and is currently alive 13 months later, in first complete remission, without sequelae, on an alleviated chemotherapy regimen.

## 1. Introduction

L-asparaginase is known to play a key role in the treatment of childhood and adult acute lymphoblastic leukemia (ALL), particularly during the induction phase [1]. This drug can lead to various and frequent adverse events, resulting in delay or cessation of chemotherapy and in morbimortality. There are no clear recommendations for their management. Age is a major risk factor for L-asparaginase toxicity, and hepatic injury is more common in adults than in children. Here, we illustrate a successful strategy overcoming multiple severe toxicities of L-asparaginase during the induction phase in a T-cell ALL (T-ALL) adult patient.

## 2. Case Presentation

T-ALL was diagnosed in a 51-year-old man, with no medical history but obesity (BMI = 30.9 kg/m^2^). He had mediastinal, pleural, pericardial, bone and pancreatic dissemination, no central nervous system involvement, normal karyotype, and high-risk NRAS mutation associated to FBXW7 and NOTCH1 mutations.

**Figure 1:**
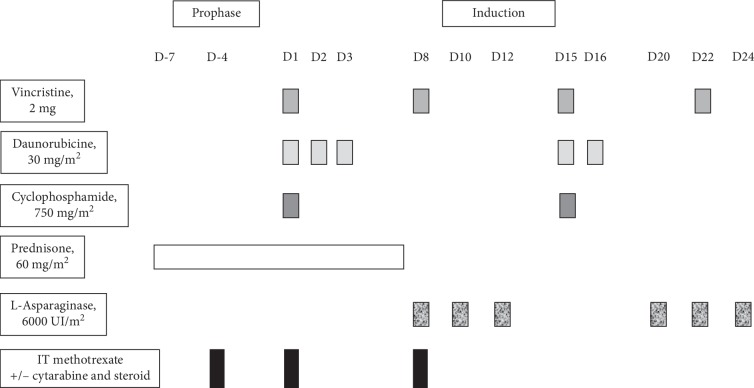
Prophase and induction phase of the GRAALL-2014 protocol for patients aged >45 years.

The patient was included in the GRAALL-2014 study (ClinicalTrials.gov Identifier: NCT02617004). After a seven-day prophase of steroids, the induction phase was composed of five drugs and prophylaxis of the central nervous system (CNS) involvement by repeated intrathecal injections (Figure 1). Native L-asparaginase is administered at the dose of 6000 UI/m2 on days 8, 10, 12, 20, 22, and 24. Of note, age-adapted doses of steroids, daunorubicin, and L-asparaginase are delivered to patients aged 46 to 60 years, as compared with patients aged 18 to 45 years, as a result of a high treatment-related mortality rate in the oldest patients in previous trials [2, 3].

According to the protocol, the patient received anticoagulant prophylaxis by unfractionated heparin from day 8 and was supplemented with antithrombin III if below 60%.

From day 24, cholestasis and conjugated hyperbilirubinemia appeared. Doppler ultrasonography of the liver showed previously unknown steatosis, no focal parenchymal lesion, no vascular lesion, particularly no portal thrombosis, and a discrete dilatation of intrahepatic bile ducts without any visible obstacle.

Autoimmune (antinuclear, ANCA, antimitochondria, anti-LKM1, antismooth muscle, and antiliver cytosol type 1 antibodies) and viral (detection of viremia by polymerase chain reaction of HBV, HCV, CMV, EBV, HSV 1&2, VZV, and HHV8) screenings were negative.

Transcutaneous liver biopsy was performed a few days later, showing marked steatosis and cholestasis, suggestive of drug toxicity attributable to L-asparaginase. It has to be noted that there was no concomittant medication with common potential hepatic toxicity, except for other chemotherapies such as daunorubicine and cyclophosphamide.

**Figure 2:**
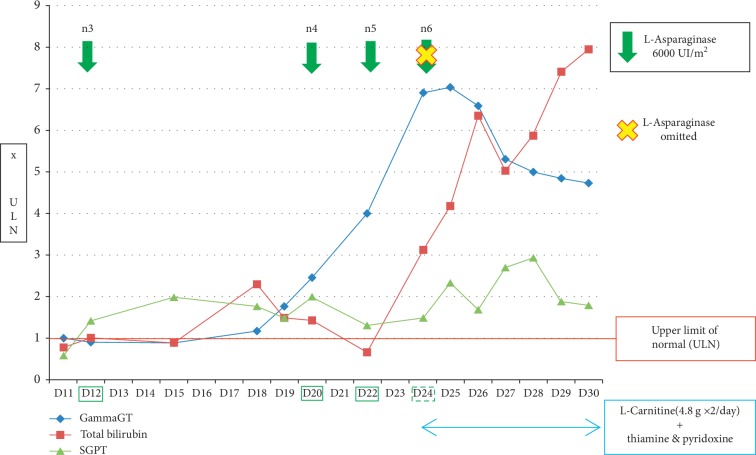
Variation of liver biologic parameters from D11 to D30 of the induction phase.

L-asparaginase was omitted on day 24, and the patient received supplementations in L-carnitine (4.8 g twice a day), thiamine (100 mg per day), and pyridoxine (250 mg per day) from this date to day 30. Evolution of hepatic parameters between D11 and D30 of the induction phase is described in Figure 2.

From day 28, headaches and nausea appeared, and vigilance progressively decreased.

On day 30, the patient was in a febrile coma, with no sign of focalization. Laboratory results revealed normal white blood cells and platelet counts. Bilirubinemia reached 7 folds, ammonemia reached 2 folds above normal value, prothrombin time was 68%, factor V 86%, and antithrombin III 46%.

Cerebral CT-scan showed cerebral venous thrombosis of the medial longitudinal sinus and diffuse cerebral edema.

This multifactorial diffuse cerebral edema was due to hepatic encephalopathy, hyperammoniemic encephalopathy, and cerebral venous thrombosis, all mechanisms that can be linked to specific toxicities of L-asparaginase.

The patient was admitted to intensive care unit on day 30.

After initiation of usual measures of neurological protection, deep sedation and intubation, curative anticoagulation by unfractionated heparin, and empirical piperacilline-tazobactam for probable aspiration pneumonia, two specific treatments were begun in order to manage hepatic and neurological deficiencies: intravenous sodium benzoate at the dose of 12 g per day was given for five days, and the patient followed daily albumin-based liver dialysis sessions of Molecular Adsorbents Recirculating System (Mars) type for 6 days.

**Figure 3:**
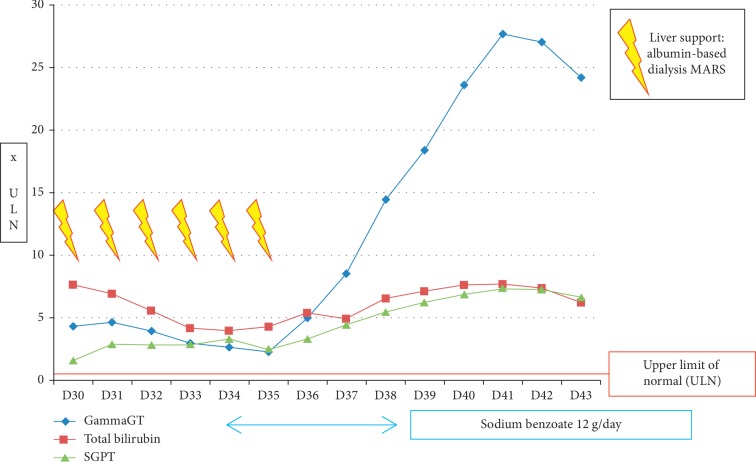
Variation of liver biologic parameters from D30 to D43 of the induction phase.

**Figure 4:**
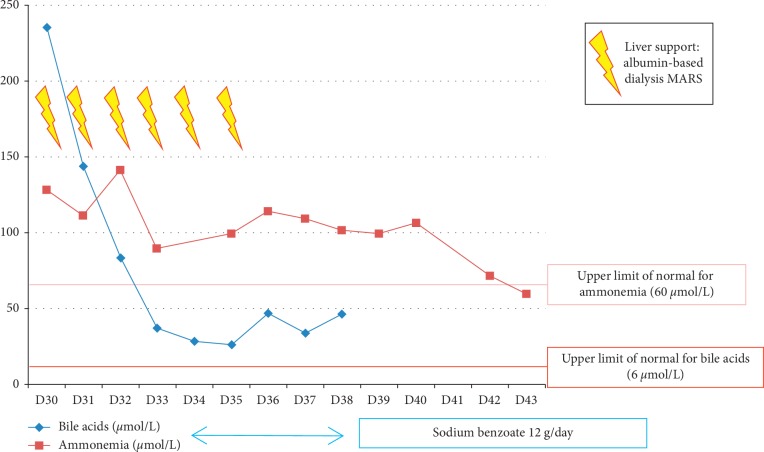
Variation of bile acids and ammonemia from D30 to D43 of the induction phase.

Hepatic biologic parameters stabilized and ammonemia progressively decreased and finally normalized. Evolution of hepatic parameters between D30 and D43 of the induction phase is described in Figure 3; variation of bile acids and ammonemia from D30 to D43 is depicted in Figure 4. Deep sedation was stopped at day 41, and consciousness improved. However, the patient's condition deteriorated at day 42 because of acute subdural hematoma. Surgery was performed, and anticoagulants were stopped. A few days later, colloid epidural patches were performed to treat intracranial hypotension syndrome, related to lumbar punctures for CNS prophylaxis.

Meanwhile, complete remission (CR) was assessed on Day 26.

Currently, the patient is in continuous remission at 13 months, with negative minimum residual disease and normal neurological and hepatic functions. He still follows the GRAALL-2014 protocol and consolidation and delayed intensification phases, with decreased dosage and frequency of chemotherapy administration. Inclusion in the ATRIALL phase 2 substudy of nelarabine-containing consolidation [4] in patients with an adverse oncogenetic profile was declined, due to the potential neurotoxicity of the drug. L-asparaginase and intrathecal injections were contraindicated for the rest of the treatment. Indeed, severe intracranial hypotension syndrome was directly linked to intrathecal injections performed during the induction phase, and it had to be treated with colloid epidural patches. Encephalic irradiation treatment has been performed to ensure CNS prophylaxis. After achievement of negative minimal residual disease at the end of induction and at midconsolidation, allogeneic bone marrow transplantation was not indicated.

## 3. Discussion

Through this case, multiple severe and specific toxicities of L-asparaginase during induction therapy of ALL are illustrated. They led to neurologic failure linked to diffuse cerebral edema.

L-asparaginase hydrolyses asparagine into L-aspartic acid and ammonia, depriving leukemic cells of this key amino acid they cannot produce by themselves. L-asparaginase has also a weak glutaminase activity. Therefore, by its own mode of action, L-asparaginase produces three neurotoxic agents: ammonia, L-aspartic acid, and glutamic acid. These two amino acids can induce cell death in CNS neurons by excessive stimulation through NMDA (*N*-methyl-D-aspartate) receptor, leading to a major intracellular calcium influx and apoptosis. In our patient, glutamic acid at the onset of coma was normal and probably did not play a key role in the neurologic deficiency. On the contrary, ammonemia was high, due to probably in part to overproduction and in part to L-asparaginase-induced hepatic failure [5]. Sodium benzoate has been given with the aim of lowering ammonemia. Indeed, sodium benzoate may bypass the purification cycle of nitrogen toward alternative metabolic pathways which do not produce ammonia. In our case, ammonemia first stabilized, before normalizing five days after the end of sodium benzoate administration.

The patient presented a grade 3 cholestasis and a grade 3 cytolysis. Hepatic toxicity of L-asparaginase is common, and increased levels of bilirubin have been reported in 30 to 60% of patients receiving it. In most cases, liver toxicity and hyperbilirubinemia are not life-threatening and resolve several weeks after drug discontinuation [6]. As recently described [7], L-asparaginase induces liver injury with short latency and a marked steatosis, probably due to direct inhibition of hepatic protein synthesis caused by asparagine depletion. In our case, cholestasis was prominent, but other abnormalities can occur, such as cytolysis, sinusoidal obstruction syndrome, or fulminant acute hepatitis for example. This toxicity is more frequent and severe in obese patients [8], like our patient. It may be linked to more frequent hepatic steatosis in obese patients at the beginning of the treatment. Obesity may also impair efficacy of L-asparaginase because of the potency of adipocytes to release glutamine, which inhibits cell cytotoxicity induced by the drug [9]. Other risk factors for grade 3-4 hyperbilirubinemia include older age and previous hyperbilirubinemia.

Facing this situation, two therapeutic approaches were proposed. The first one was supplementation in L-carnitine (along with thiamine and pyridoxine). L-carnitine is a derivate of lysine, an amino acid involved in production of energy through fatty acid oxidation. It has shown efficacy in liver protection in case of nonalcoholic steatosis, and preclinical studies in murine models concerning hepatic toxicity of chemotherapy (including L-asparaginase) have shown promising results in reducing hepatic lesions [10]. Different mechanisms of hepatic protection by L-carnitine are known or proposed: reduction of oxygen consumption in steatotic livers and reduction of vascular resistance in the portal system. L-asparaginase induces mitochondrial dysfunction caused by amino acid depletion, resulting in accumulation of unoxidized fatty acids which are directly responsible for hepatic damages. L-carnitine, through its roles in fatty acids metabolism, helps to maintain normal mitochondrial function and cell viability under these abnormal cellular conditions [11]. Few clinical experiments of L-carnitine in management of hepatic toxicity of L-asparaginase exist, showing some cases of improvement of the liver enzyme counts [12], but there is no prospective or comparative study to affirm the efficacy of L-carnitine in this situation. Our second therapy for liver failure was dialysis with molecular adsorbent recirculating system (Mars). This treatment is currently indicated in severe hepatic failure with encephalopathy and often represents a “bridge-to-liver transplantation” in cirrhotic patients in intensive care units [13]. It replaces liver functions of purification of bilirubin, bile acid, ammonia, and several cytokines (nitric oxide, IL6, TNF*α* etc). In our patient, daily sessions for six days have been performed. Bile acid level decreased rapidly, ammonia more slowly. To our knowledge, it is the first report of the use of Mars to manage hepatic toxicity of chemotherapy. Although the role of each component of this integrative strategy is difficult to assess, sodium benzoate and Mars dialysis seem to have significantly contributed to the favorable biological and clinical outcome, with complete neurological recovery and ultimately successful treatment of ALL.

In this case, the native form of L-asparaginase was used. Other forms of L-asparaginase exist in the induction therapy of ALL, such as pegylated asparaginase. Compared with native L-asparaginase, pegylated form shares the same toxicity profile and does not seem less toxic in adult patients [14, 15]. Indeed, more than a third of the patients face grade 3 to 5 hepatic toxicity with pegylated asparaginase.

Daunorubicin was the main concomitant medication with a common hepatotoxicity. It may have played a role in the liver dysfunction in our patient since this drug is known to have a direct hepatic toxicity. The hepatic injury due to daunorubicin varies in severity from mild, transient, and asymptomatic liver enzyme elevations to acute liver failure due to sinusoidal obstruction syndrome. Nevertheless, the timing, type, and severity of the liver dysfunction indicated that it was more attributable to L-asparaginase.

## 4. Conclusion

Neurological deficiency under L-asparaginase chemotherapy for ALL may result from intricated toxicities. Albumin-based dialysis (Mars) and supplementation in L-carnitine, thiamine, and pyridoxine should be discussed in case of hepatic encephalopathy, along with sodium benzoate to limit hyperammonemia. We think that this innovative approach is warranted in front of life-threatening L-Asparaginase toxicities.
